# Fault Diagnosis for Micro-Gas Turbine Engine Sensors via Wavelet Entropy

**DOI:** 10.3390/s111009928

**Published:** 2011-10-21

**Authors:** Bing Yu, Dongdong Liu, Tianhong Zhang

**Affiliations:** School of Energy and Power Engineering, Nanjing University of Aeronautics and Astronautics, Nanjing 210016, China; E-Mails: liudongdong_fadec@yeah.net (D.L.); thz@nuaa.edu.cn (T.Z.)

**Keywords:** fault diagnosis, wavelet entropy, wavelet decomposition, gas turbine sensor

## Abstract

Sensor fault diagnosis is necessary to ensure the normal operation of a gas turbine system. However, the existing methods require too many resources and this need can’t be satisfied in some occasions. Since the sensor readings are directly affected by sensor state, sensor fault diagnosis can be performed by extracting features of the measured signals. This paper proposes a novel fault diagnosis method for sensors based on wavelet entropy. Based on the wavelet theory, wavelet decomposition is utilized to decompose the signal in different scales. Then the instantaneous wavelet energy entropy (*IWEE*) and instantaneous wavelet singular entropy (*IWSE*) are defined based on the previous wavelet entropy theory. Subsequently, a fault diagnosis method for gas turbine sensors is proposed based on the results of a numerically simulated example. Then, experiments on this method are carried out on a real micro gas turbine engine. In the experiment, four types of faults with different magnitudes are presented. The experimental results show that the proposed method for sensor fault diagnosis is efficient.

## 1. Introduction

Gas turbines are one of the most important types of power equipment, widely used in modern aircraft propulsion systems and power systems. In order to achieve their challenging customer-driven performance requirements, gas turbines have to operate at their physical limits, which are set by material and gas properties. However, the normal operations of the gas turbines rely on the fine tuned cooperation of many different components, and any minor problem with these components may adversely affect the gas turbine system. Thus, advanced controls and monitoring technologies to improve the safety and reliability of the gas turbine system are widely studied, and at the same time, to reduce the operating cost.

The control and monitoring of gas turbine systems depend on accurate and reliable sensor readings from a considerable number of sensors. All these sensors must be functional to ensure normal operation of a gas turbine, otherwise, sensor failures may mislead the controller, and lead to the paralysis of the entire system. Therefore, in order to improve the reliable operation of systems, it is necessary to employ sensors fault diagnosis in a timely and accurate fashion.

For the reasons described above, sensor fault diagnosis has received considerable attention and many works on this topic have been reported. Isermann [1] presented an extended review of the field of fault detection and diagnosis, in which many fault detection and diagnosis methods were presented. Botros *et al.* [2] proposed an application of radial basis function neural networks for sensor fault detection on an RB211 gas turbine driven compressor station. Napolitano *et al.* [3] discussed the performance of a simulated neural network based fault-tolerant flight control system for sensor fault detection. Dewallef and Leonard [4] worked out a methodology for on-line sensor validation in turbojet engines using neural networks for generation of the mathematical representation of the engine. Ogaji *et al.* [5] reported a multiple sensor fault diagnoses method for a 2-shaft stationary gas turbine. Romesis *et al.* [6] presented a sensor fault detection method based on a probabilistic neural network. Aretakis *et al.* [7] reported an approach for detecting sensor faults in turbofan engines. Mehranbod *et al.* [8] presented a Bayesian belief network (BBN)-based sensor fault detection and identification method. Palmé *et al.* [9] also presented recently a similar solution for sensor validation based on artificial neural networks.

With increasing demand on small power systems and small propulsion systems, many micro gas turbines are being developed, and excellent success has been achieved in this area. However, the power to weight ratio of a micro-gas turbine should be high, so its EEC (electronic engine controller) should be light and have low power consumption, thus the microcontroller in the EEC is not powerful enough to adopt nonlinear engine model or neural networks for sensor validation. Consequently, the previous approaches are not suitable for micro-gas turbines. To overcome such problems, a simple and efficient approach should be adopted.

Typically, there are five types of sensor faults: the step fault (including the open and short fault), the pulse fault, the periodic fault, the noise fault and the drift fault. If any type of fault occurs, except for the drift fault, transient readings from the sensor will be affected. Thus, the fault status of the sensor could be extracted by analyzing the sensor readings. A mature and efficient tool for signal analysis is Fourier analysis, which transforms the sensor signal from a time-based domain to a frequency-based one. However, it is hard to determine when a particular fault takes place. To improve this deficiency, Gabor proposed the short-time Fourier transform (STFT) [10]. The windowing technique analyzes a small section of signal each time. The STFT maps a signal into a 2-D function of time and frequency. Still, the transformation has disadvantage that the time or frequency information can only be obtained with limited precision, which is determined by the size of the window. A higher resolution in both time and frequency domain cannot be achieved simultaneously since once the window size is fixed, which is the same for all frequencies. The wavelet transform (WT) is a popular technique to analyze signals. Wavelet functions are composed of a family of basis functions that are capable of describing a signal in both the time and frequency domains [11]. To enhance the performance of the wavelet transform, the concept of wavelet entropy is introduced in feature extraction. The method is widely used in many fields, and has achieved considerable success [12–16].

This paper proposes a fault diagnosis method for micro-gas turbine sensors operating under nonstationary conditions. The fault types mentioned above are investigated, except for drift faults. Based on wavelet theory, wavelet decomposition is utilized to decompose the signal in different scales. Then the instantaneous wavelet energy entropy (*IWEE*) and instantaneous wavelet singular entropy (*IWSE*) are defined based on the previous wavelet entropy theory. Subsequently, a fault diagnosis method for gas turbine sensors is proposed based on the numerically simulated example. Finally, experiments are carried out on this method, and the results show that it is efficient.

## 2. Theoretical Background

### 2.1. Wavelet Transform *[11]*

Wavelets are finite-energy functions with good localization properties in both the frequency and time domain, which can be used very efficiently to represent transient signals. For a given mother wavelet *ψ*(*t*), a scaled and translated version (wavelet family) is designated by:

(1) $$\psi_{a,b}={\left|a\right|}^{-\frac{1}{2}}\psi (\frac{t-b}{a})$$

where the parameter *a* corresponds to the scale, while *b* is the translation parameter, and *t* stands for time.

For a given signal *f*(*t*) ∈ *L*^2^(ℝ), the results of the wavelet transform are the correlation between the function *f(t)* with the family wavelet *ψ*_*a,b*_ for each *a* and *b*:

(2) $$W_{f}\;(a,b)=\int_{R}f(t)\bar{\psi_{a,b}(t)}dt={\left|a\right|}^{-\frac{1}{2}}\int_{R}f(t)\bar{\psi (\frac{t-b}{a})}dt$$

By selecting a special mother wavelet function ψ(*t*) and the discrete set of parameters (*a_j_* *= 2^−j^*, *b_j,k_ = 2^−jk^*, *j,k* ∈ *Z*), the wavelet family could be presented as:

(3) $$\psi_{j,k}(t)=2^{\frac{j}{2}}\psi (2^{j}t-k),\;j,k\in Z$$

The discrete wavelet transform (DWT) of signal *f*(*t*) can be obtained through Equation (4).

(4) $${\mathrm{DWT}}_{f}\;(j,k)=\int_{R}f(t)\psi_{j,k}(t)dt=2^{-\frac{j}{2}}\int_{R}f(t)\psi (2^{-j}t-k)dt$$

### 2.2. Wavelet Decomposition and Wavelet Entropy

In 1989 Mallat presented a fast wavelet decomposition algorithm for discrete wavelet transform, which utilizes the orthogonal wavelet bases to decompose the signal under different scales [17]. It is equivalent to recursively filtering a signal with a high-pass and low-pass filter pair which gives the detailed components and approximation components, respectively.

By using Mallat’s mathod, a certain time serise *x*(*k*) (*k = 1, 2,…, N*) could be decomposed as:

(5) $$x(k)=A_{1}(k)+D_{1}(k)=A_{2}(k)+D_{2}(k)+D_{1}(k)=A_{J}\;(k)+\sum_{j=1}^{j=J}D_{j}(k)$$

where *j* represents scale, *D_j_*(*k*) represents the detailed components at the *j*th scale and *A_J_*(*k*) represents the approximation of the Jth scale.

The frequency band of *A_j_*(*k*) and *D_j_*(*k*) could be represented by:

(6) $$\{\begin{array}{c} D_{j}\left(k\right):\left[2^{-(j+1)}f_{s},2^{-j}f_{s}\right]\\ A_{j}\left(k\right):\left[0,2^{-\left(j+1\right)}f_{s}\right] \end{array}\;(j=1,2,\ldots ,J,k=1,2,\ldots ,N)$$

where *fs* is the sampling frequency.

Since the wavelet bases used to decompose the signal are orthogonal, these decomposed signals could be regarded as a direct estimation of local energies at different scales [17]. Thus, the wavelet engery of detailed components at instant k and scale j will be represented as:

(7) $$E_{j,k}={\left|D_{j}(k)\right|}^{2},j=1,2,\ldots ,J,k=1,2,\ldots ,N$$

For the purpose of unification, the wavelet engery of approximation components at instant *k* and scale *J* is defined as:

(8) $$E_{J+1,k}={\left|A_{J}\left(k\right)\right|}^{2},k=1,2,\ldots ,N$$

Consequently, the wavelet engery at each sacle (J + 1 scale means approximation components) could be described as:

(9) $$E_{j}=\sum_{k=1}^{N}E_{j,k}\;,j=1,2,\ldots ,J+1$$

The total wavelet engery could be defined as:

(10) $$E_{\mathrm{tol}}=\sum_{j=1}^{J+1}E_{j}$$

### 2.3. Wavelet Entropy

The concept of entropy is derived from thermodynamic entropy, which can be roughly seen as a measure of the degree of system chaos. In the information world, Shannon defined the Shannon entropy that can represent the degree of chaos of a system [18]. It provides an efficient criterion for analyzing and comparing probability distributions. Given a random variable *X* which takes a finite number of possible values *x*_1_, *x*_2_, …, *x_n_* with probabilities *p*_1_, *p*_2_, …, *p_n_* respectively, where *p_i_* ≥ 0, *i* = 1,2, …, *n* and $\sum_{i=1}^{n}p_{i}=1$. The Shannon entropy could be written as:

(11) $$H(X)=-\sum_{i=1}^{n}p_{i}\;\text{log}\left(p_{i}\right)$$

The concept of wavelet entropy is inherited from Shannon entropy. Many types of wavelet entropies have been defined to solve different problems, and these methods can achieve good detection and recognition performance. Blanco *et al.* [12] defined wavelet entropy based on wavelet transform in 1998 and applied it to analyse electroencephalography traces. He *et al.* [13] defined several kinds of wavelet entropy to analyse transient signals in power systems. Ren *et al.* [16] proposed a method for structural damage identification via wavelet entropy.

The wavelet entropy proposed above can represent the degree of order/disorder of the measured signal from sensors, which can provide useful information about the underlying state of the sensors. However, in critical circumstanced, such as gas turbine operation, sensor faults should be detected immediately. Gathering a large number of sensor readings and performing a post-analysis is not practical. Thus, it is necessary to define a type of instantaneous wavelet entropy to solve this problem. Based on the definition in [13], the instantaneous wavelet energy entropy (*IWEE*) and instantaneous wavelet singular entropy (*IWSE*) are defined as follows.

To obtain the instantaneous wavelet entropy of *x*(*n*) at instant *k*, a window of the time series is picked out, *i.e., x_w_*(*n*) = *x*(*k* − *W* + 1),…, *x*(*k*), *k* − *W* + 1 > 0, where *W* is the width of the window. More information could be obtained when a higher vaule of *W* is chosen, however, this implies more calculation costs, thus, the proper value of *W* should be considered. Decomposing *x_W_*(*n*) via wavelet decomposition, then a series of signal at different scale could be produced as:

(12) $$x_{W}\;\left(n\right)=A_{\mathrm{WJ}}\;\left(n\right)+\sum_{j=1}^{J}D_{\mathrm{Wj}}\;(n),n=k-W+1,\ldots ,k$$

As defined in Equation (5) the wavelet energy of *x_W_*(*n*) at each scale and total could be represented as in Equations (13) and (14):

(13) $$E_{\mathrm{Wj}}=\sum_{n=k-W+1}^{k}{\left|A_{\mathrm{WJ}}\;\left(n\right)\right|}^{2}+\sum_{j=1}^{J}\sum_{n=k-W+l}^{k}{\left|D_{\mathrm{Wj}}\;(n)\right|}^{2}=\sum_{n=k-W+1}^{k}E_{Wj,n},\;j=1,2,...,J+1$$

(14) $$E_{\mathrm{Wtol}}=\sum_{j=1}^{J+1}E_{\mathrm{Wj}},\;j=1,2,...,J+1$$

Thus, we define the normalized *p_j_*, with *p_j_ = E_Wj_*/*E_Wtol_, j* = 1,2, …, *J* + 1 and ∑*_j_p_j_* = 1, which represents the distribution of wavelet energy at different scales. According to Equation (11), the *IWEE* at instant *k* will be defined as:

(15) $$S_{\mathrm{IWEE}}(k)=-\sum_{j=1}^{J+1}p_{j}\;\text{log}\left(p_{j}\right)$$

As mentioned above, the approximation components and detailed components could be obtained through wavelet decompositions, and the detailed componets contain high frequency information of the original siganl. As we all know, a mutation will occur in the measured sensor signal when the sensor fails, and this mutational signal lies in the detailed components certainly. Thus, the *IWSE* is defined based on the detailed components, which aims to uncover the fault information contained in the sensor signal.

As described in Equation (12), detailed components of the decomposed signal can constitute a *W* × *J* matrix D, where *W* is the width of the slide window and *J* means scale number. According to the singular value decomposition theory, there exist a *W* × *l* matrix U, an *l* × *J* matrix V and an *l* × *l* matrix Λ, which could make the relationship given in Equation (16):

(16) $$D_{W\times J}=U_{W\times l}\Lambda_{l\times l}V_{l\times J}^{T}$$

The diagonal elements *λ_i_*(i = 1,2,…,*l*) of the diagonal matrix Λ are not negative and in descending order. These diagonal elements are the singular values of the matrix D. As described in the signal singular value decomposition theory, when the signal has no noise or high signal-to-noise ratio, only a few diagonal elements exist. Thus, these diagonal elements could be employed to extract information about the frequency distribution at different scales, so a variable *q_j_* is defined as Equation (17), where *q_j_* > 0, ∑*_l_q_j_* = 1, *j* = 1, 2, …, *l*, and the *IWSE* at instant *k* is defined as Equation (18):

(17) $$q_{j}=\lambda_{j}/\sum_{j=1}^{l}\lambda_{j}$$

(18) $$S_{\mathrm{IWSE}}\;(k)=-\sum_{j=1}^{l}q_{j}\;\text{log}\left(q_{j}\right)$$

## 3. Sensor Fault Diagnosis Method Based on Wavelet Entropy

### 3.1. Numerically Simulated Example

To illustrate the fault diagnosis ability by the proposed *IWWE* and *IWSE*, the following numerical simulations are investigated. Given a stationary signal *S*(*t*) = (2 + *N*(0,0.04))*V* (0 ≤ t < 10s), where N(0,0.04) is Gaussian white noise with covariance 0.04, which stands for the stationary signal from a normal sensor. To represent the signal with faults, several kinds of fault signals, such as pulse fault, noise fault, periodic fault and step fault signals, are added in to *S*(*t*), then the new siganl *f*(*t*) could be represented as:

(19) $$f(t)=\left\{ \begin{array}{l} S(t),(0\leq t<1s)\\ S(t)+0.6,\left(1\leq t<1.05s\right)\\ S(t),\left(1.05\leq t<2s\right)\\ S(t)+N(0,0.6),\left(2\leq t<4s\right)\\ S(t),(4\leq t<5s)\\ S(t)+0.6\;\text{sin}(\pi t/2),(5\leq t<7s)\\ S(t),\left(7\leq t<8s\right)\\ S(t)+0.6,(8\leq t<10s) \end{array}\right.$$

**Figure 1.**
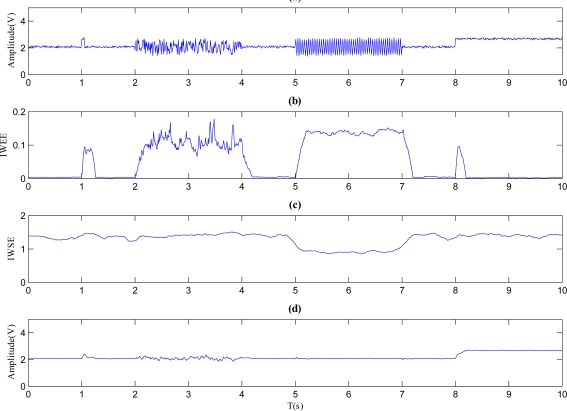
Results of the numerical simulations. **(a)** Signal with four kinds of faults. **(b)** *IWWE* of the siganl. **(c)** *IWSE* of the signal. **(d)** *A_mean_* of the signal.

Sampling *f*(*t*) with a frequency of 100 Hz, a discrete verison of signal *f*(*n*) can be produced. Let the mother wavelet type be ‘*haar*’ wavlet (requiring less calculation), *W = 20* and *J = 5*, then the time series *f*(*n*) is decomposed, at the same time, the *IWEE* and *IWSE* are calculated. The corresponding results are shown in Figure 1. Figure 1(a) represents the discrete time series *f*(*n*), while Figure 1(b,c) stand for the *IWEE* curve and *IWSE* curve separately. Figure 1(d) presents the curve of mean value of approximation components at *J* scale. According to Equation (12), the mean value of approximation components at *J* scale at instant *k* can be represented as:

(20) $$A_{\mathrm{mean}}\;(k)=\sum_{n=k-W+1}^{k}A_{\mathrm{WJ}}\;\left(n\right)/W,n=k-W+1,\ldots ,k$$

As shown in Figure 1(b), the *IWEE* value mutates and grows where the fault signals are mixed in, which can indicates when the faults occur. Meanwhile, the *IWEE* value changes and returns to normal quickly when a pulse or step fault occurs. However, the *IWEE* value changes and stays for a while when a noise or periodic fault occurs. Since the *IWEE* value pattern when a pulse or step fault occurs is identical, the *A_mean_*(*n*) could be employed to distinguish them as shown in Figure 1(d). The *A_mean_*(*n*) will be changed when a step fault occurs. Similarly, noise or periodic faults could be distinguished through *IWSE*. As Figure 1(c) shows, the *IWSE* value goes low when a periodic fault happens, however it is steady when a noise fault occurs. As the result of numerically simulated example shown, the proposed *IWEE* and *IWSE* are good tools for fault diagnosis.

### 3.2. Proposed Method for Sensor Fault Diagnosis Based on Wavelet Entropy

**Figure 2.**
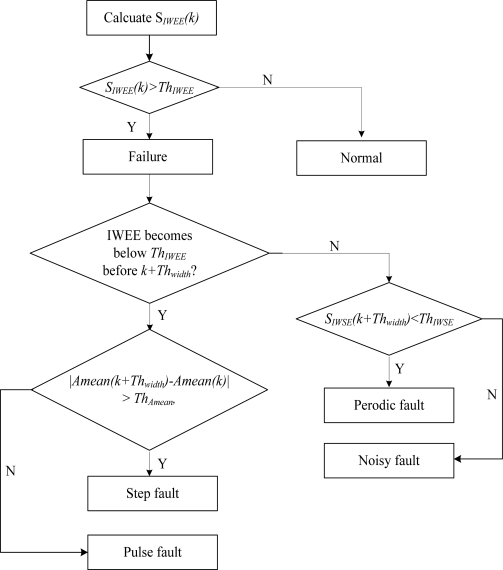
Flowchart of proposed sensors fault diagnosis method.

The sensor fault diagnosis method is proposed based on the results of the numerical simulations. In order to explain the method clearly, several parameters are defined in Table 1. The proposed sensor fault diagnosis method is shown in Figure 2.

Step 1. Combine the sensor reading at time *k* with the *W*−1 samples gathered previously, then a new time series with the length *W* could be obtained, which is presented as *x*(*n*), (*n* = *k* − *W* + 1, …, *k*).
Step 2. Figure out the *IWEE* of x(n) and compare it with the *Th_IWEE_*, the sensor is judged to be faulty when *IWEE* is more than *Th_IWEE_*, otherwise the sensor is normal.
Step 3. Monitor the value of *IWEE*, if it becomes less than *Th_IWEE_* before time *k + Th_width_* is reached, the fault is a pulse or step type fault (CASE 1), otherwise, the fault type should be a noise or periodic fault (CASE 2). At the same time, calcuate the *A_mean_* and *IWSE* from time *k* to *k + Th_width_*.
Step 4. For CASE 1 in Step 3, check if S*_IWSE_* (*k + Th_width_*) < *Th_Amean_*, if yes the fault type is noise, otherwise the fault type is periodic. For CASE 2 in Step 3, check if *| A_mean_(k + Nd) − A_mean_(k)| > Th_Amean_*, if yes the fault type is step, otherwise the fault type is pulse.

## 4. Experiments on a Micro Gas Turbine Engine

**Figure 3.**
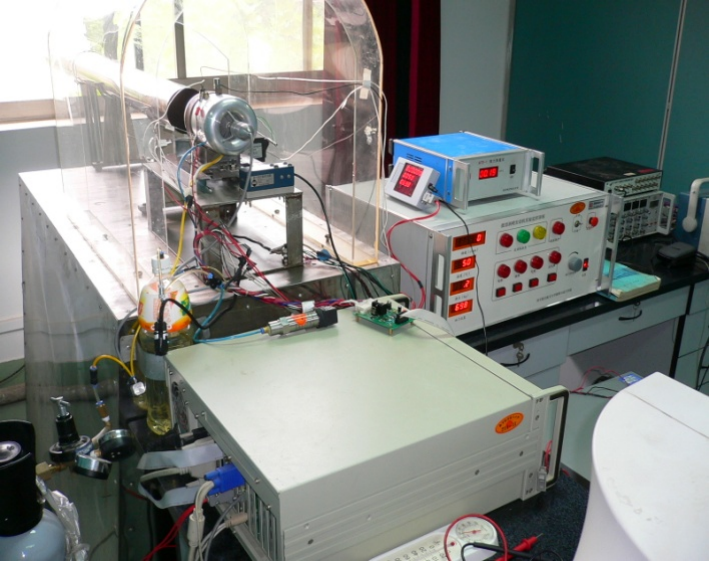
Test environment for the experiments.

The experiments for the proposed method are carried out on the “Chao Ying” engine, which is a single shaft micro-gas turbine developed at the Nanjing University of Aeronautics and Astronautics. The temperature sensor placed in the nozzle is employed to verify the proposed sensor fault diagnosis method. In our system, the temperature sensor is made from a platinum thermocouple, and the temperature range that can be sensed by it is from 0 to 1,600 °C, and the output of the sensor is adjusted to 0 to 5 V. The environment for experiments is shown in Figure 3. To carry out the experiments, the output of the temperature sensor is sampled and then forwarded to a PC through a RS232 serial port. To simulate the output of a sensor with fault, various types of faults were artificially added using software.

In this experiment, the main parameters for the method are chosen as listed in Table 2. In order to demonstrate the performance of the proposed method in a practical situation, the micro-gas turbine engine is set to work under nonstationary conditions by adjusting the angle of the throttle lever. Thus, the the measured signal of the sensor is dynamic and similar to what would be encountered under practical conditions. To form fault signals artificially, four types of faults (pulse, step, noise and periodic) are added into the normal time series separately.

Considering minimum detectable fault magnitude (MDFM) is an important parameter for the proposed method, and it is also a significant index of practicality. Henceforth, the minimum detectable fault magnitude for the proposed method should be determined through the experiment, so in the following experiment, four types of faults with 1%, 5%, 10% and 15% magnitude variations are presented. The detailed analysis is as follows.

**Figure 4.**
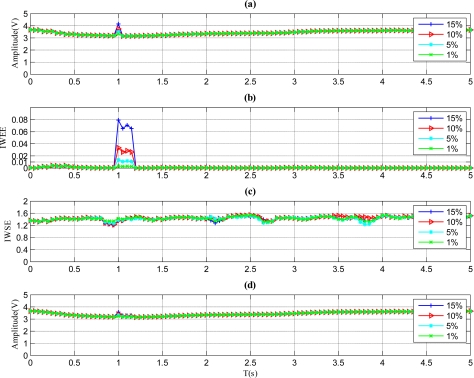
Results for the sensor with pulse faults. **(a)** Signal of sensor with pulse faults. **(b)** *IWWE* of the signal. **(c)** *IWSE* of the signal. **(d)** *A_mean_* of the signal.

The results of a sensor with a pulse fault are described in Figure 4. As shown in Figure 4(b), the *IWEE* value grows higher than *Th_IWEE_* when the fault magnitude is 5%, 10% or 15%. However, the IWEE value is below *Th_IWEE_* when the fault magnitude is 1%, thus the fault with 1% fault magnitude is undetectable through the proposed method. Therefore, the MDFM for this kind of fault is regarded as 5%. Furthermore, the *A_mean_* value in Figure 4(d) barely varies when the faults occur, so the fault type is determined to be pulse, which is identical to Figure 2.

**Figure 5.**
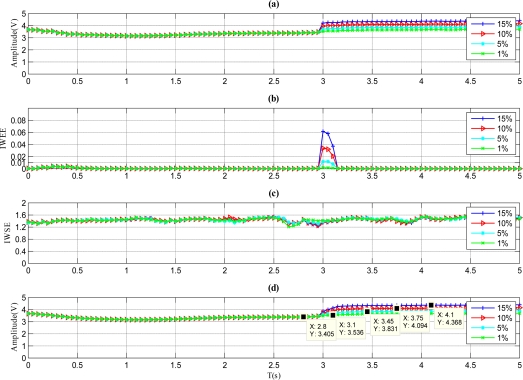
Results for the sensor with step faults. **(a)** Signal of sensor with step faults. **(b)** *IWWE* of the signal. **(c)** *IWSE* of the signal. **(d)** *A_mean_* of the signal.

The results of a sensor with step fault are illustrated in Figure 5. Like Figure 4(b), the *IWEE* value of each magnitude variation grows when the faults occur in Figure 5(b). In Figure 5(d), the increased *A_mean_* value is more than *Th_Amean_* when the fault signal amplitude is 5%, 10% or 15%, so the fault type is identified to be step according to Figure 2. However, the increased *A_mean_* value is lower than *Th_Amean_* when the fault signal amplitude is 1%, so the MDFM for the step fault is also set at 5%.

**Figure 6.**
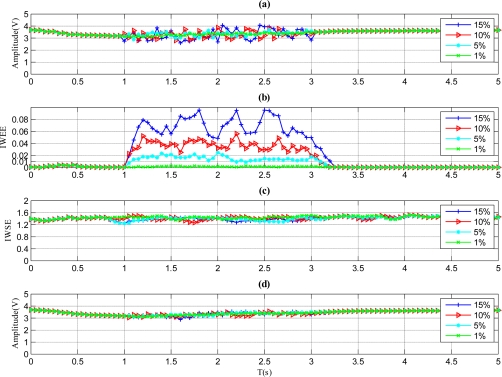
Results for the sensor with noise faults. **(a)** Signal of sensor with noise faults. **(b)** *IWWE* of the signal. **(c)** *IWSE* of the signal. **(d)** *A_mean_* of the signal.

The results of sensor with noise fault are illustrated in Figure 6. As shown in Figure 6(b), the *IWEE* value of each magnitude variation grows and remains until the fault disappears. For this duration, the *IWEE* value stays higher than *Th_IWEE_* when the fault magnitude is 5%, 10% or 15%, but is less than *Th_IWEE_* when the fault magnitude is 1%, so the fault with 1% fault magnitude is undetectable by the proposed method, and the proper MDFM for the this fault type should be 5%. As to the *IWSE* shown in Figure 6(c), the *IWSE* values for all magnitude variations stay higher than *Th_IWSE_*, thus the fault type is identified to be noise, according to Figure 2.

**Figure 7.**
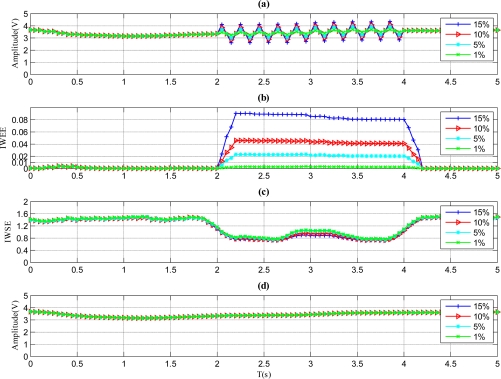
Results for the sensor with pulse faults. **(a)** Signal of sensor with pulse faults. **(b)** *IWWE* of the signal. **(c)** *IWSE* of the signal. **(d)** *A_mean_* of the signal.

Figure 7 represents the situation for a periodic fault. As shown in Figure 7(b), the *IWEE* value of each magnitude variation goes higher than *Th_IWEE_* and stays there until the faults recover when the fault magnitude is 5%, 10% or 15%, while it remains lower than *Th_IWEE_* when the fault magnitude is 1%. Therefore, the fault with 1% fault magnitude is undetectable by the method, and the proper MDFM for this fault type is 5%. As shown in Figure 7(c), the *IWSE* values of all situations become lower than the fault duration, so the fault type is identified to be periodic according to Figure 2.

## 4. Conclusions

The status of a sensor can be reflected by its output signal, hence it is feasible to perform sensor fault diagnosis by analysing the measured sensor signals. This paper proposes a method for sensor fault diagnosis via wavelet entropy. Based on the wavelet theory, two types of wavelet entropies, *IWEE* and *IWSE*, are defined to extract the inherent sensor information. Then a method using of *IWEE* and *IWSE* as criteria is presented. In order to verify the performance of the proposed method, an experiment is carried out on an actual micro-gas turbine engine. In the experiment, four types of faults with different magnitudes are presented. The results show that the proposed method is efficient for senor fault diagnosis.

The proposed sensor fault diagnosis method has some limitations that require improvement. Firstly, the types of sensor faults verified are limited, and more types of sensor faults should be tested via the method. Meanwhile, the values in Table 2 are chosen for particular sensor, which should be verified by more practical data. Finally, the proposed method is guaranteed to work only when the amplitude of fault signal is more than 5% of full scale, so some improvements must be done to enhance its performance.

## Figures and Tables

**Table 1.** Parameters for proposed method.

**Parameters**	Discription
*Th_IWEE_*	The threshold of *IWEE* value to ckeck if any fault exists.
*Th_width_*	The threshold to distinguish if the mutation of ***IWEE*** value keeps higher than *Th_IWEE_* for a certain time or not.
*Th_IWSE_*	The threshold of *IWSE* value to distinguish whether the fault type is noise or perodic.
*Th_Amean_*	The threshold of changed value of *A_mean_* to distinguish whether the fault type is step or pulse.

**Table 2.** Chosen parameters values for the experiments.

**Parameters**	**Value**
*Sample frequency*	100 Hz
*W*	20
*J*	5
*Mother wavelet*	‘Haar’
*Th_IWEE_*	0.01
*Th_width_*	60
*Th_IWSE_*	1.15
*Th_Amean_*	0.3
